# Child diet and health outcomes of the simple suppers program: a 10-week, 2-group quasi-experimental family meals trial

**DOI:** 10.1186/s12889-019-7930-7

**Published:** 2019-12-10

**Authors:** Carolyn Gunther, Catherine Rogers, Christopher Holloman, Laura C. Hopkins, Sarah E. Anderson, Carla K. Miller, Kristen A. Copeland, Jamie S. Dollahite, Keeley J. Pratt, Alison Webster, Allison N. Labyk, Christine Penicka

**Affiliations:** 1 Department of Human Sciences, The Ohio State University, 1787 Neil Ave, 313 Campbell Hall, Columbus, OH 43210 USA; 2 Present Address: Department of Nutrition, Case Western Reserve University, 10900 Euclid Ave, Wood Building, Cleveland, OH 44106 USA; 3 Department of Statistics, The Ohio State University, Columbus, USA; 4 Present Address: Information Control Company (ICC), 2500 Corporate Exchange Dr, Columbus, OH 43231 USA; 5 Division of Epidemiology, The Ohio State University, 1841 Neil Avenue, Cunz Hall, Columbus, OH 43210 USA; 6 Division of General and Community Pediatrics, Department of Pediatrics, Cincinnati Children’s Hospital Medical Center, University of Cincinnati, 3333 Burnet Avenue, Cincinnati, OH 45229 USA; 7 Division of Nutritional Sciences, Cornell University, 408 Savage Hall, Ithaca, NY 14853 USA; 8 Present Address: Food Directions, 1101 K St NW #650, Washington, DC 20005 USA; 9 Present Address: Albany Medical Center, 43 New Scotland Ave, Albany, NY 12208 USA

**Keywords:** Family meals, Childhood obesity, Food preparation skills, Racial minority, Blood pressure

## Abstract

**Background:**

Racial minority children, particularly from low-income households, are at risk for obesity. Family meals have a protective effect on child nutritional health. However, the current evidence is limited in racial and socioeconomic diversity. The objective of this study was to evaluate the impact of a family meals intervention, Simple Suppers, on improvements in diet and health outcomes from baseline (T0) to post-intervention (T1) in intervention compared to waitlist control participants, and determine retention of change in outcomes among intervention participants at 10-week follow-up (T2).

**Methods:**

Simple Suppers was a 10-week family meals intervention implemented as a 2-group quasi-experimental trial. Ten 90-min lessons were delivered weekly. Data were collected at T0 and T1, and from intervention participants at T2. Participants were racially diverse 4–10 year-old children from low-income households. Setting was a faith-based community center. Main outcomes were daily servings of fruit, vegetables, and sugar-sweetened beverages and diet quality; z-scores for body mass index (BMI), waist circumference, systolic and diastolic blood pressure (BP); weight status categories; food preparation skills; and family meals (frequency of dinner, breakfast, TV viewing during meals, meals in dining area). Generalized linear mixed models (GLMMs) and mixed-effects ordinal regression models were used to assess intervention impact (T0:T1). Paired t-tests examined retention of change among intervention participants (T1:T2).

**Results:**

One hundred forty children enrolled and 126 completed T1 (90% retention); 71 of 87 intervention participants completed T2(79% retention). Mean (SD) age was 6.9(1.9) yr, 62% female, 60% Black, and 42% low-income. Intervention vs waitlist controls had higher food preparation skills (*p* < 0.001) and lower TV viewing during meals (*p* = 0.04) at T1.There were no group differences in dietary intake or quality or z-scores for BMI, waist circumference, or BP, however intervention versus waitlist controls experienced a greater change toward healthy weight (p = 0.04) At T2, intervention participants demonstrated a retention of improved food preparation skills.

**Conclusions:**

Simple Suppers led to improvements in children’s weight status, food preparation skills, and TV viewing during meals, but not diet or z-scores for BMI, waist circumference, or BP. Future research should examine the preventive effects of healthy family mealtime routines in children at greatest risk for obesity.

**Trial registration:**

NCT02923050; Simple Suppers Scale-up (S3); Retrospectively registered on Oct 2016; First participant enrolled on Jan 2015.

## Background

Childhood obesity is a persisting public health threat with 18.5% of US youth (2 to 19 years old) currently classified as obese [1]. Of particular concern, fluctuating rates have been identified in certain age groups of children. For instance, from 2005 to 2006 to 2013–2014, there was a positive trend among preschoolers (2 to 5 years old) and young children (6 to 11 years old), i.e., a sustained plateau, however a recent report indicates a rebound [2]. In addition, racial minority children, particularly those from low-income households, are at relatively high risk for obesity [3]. Due to the negative short- and long-term effects of obesity on children’s health, well-being [4], and academic success [5], this represents a significant public health concern and points to the urgent need for effective childhood obesity prevention interventions, especially in these populations.

The American Academy of Pediatrics recommends participation in family meals as a childhood obesity prevention strategy due to evidence demonstrating a protective effect of participation in healthy mealtime routines on child diet and weight status [6]. Family meals have been associated with increased intake of fruit and vegetables and reduced child intake of sugar-sweetened beverages as well as reduced risk of childhood obesity [7–15]. However, the current evidence linking family meals with improved child dietary intake and weight status has limitations. The majority of the family meals literature – specifically in the area of childhood obesity prevention – are observational studies, demonstrating mixed evidence of an association between family meals and child diet and weight status [16–21]. The existing family meals *intervention* research is strong with regards to study design (i.e., randomized controlled trial), however limited in number and target population (i.e., focuses primarily on White children from well-educated families) [22–25]. In addition, the majority of the current family meals research fails to examine health outcomes beyond body mass index (BMI). Inclusion of blood pressure (BP) [26] would be particularly critical as hypertension in children is associated with obesity and onset of premature diabetes [10, 27–29], even in very young children (i.e., < 5 years old) [30, 31]. There is need for additional research on family meals, specifically experimental studies with expanded health outcomes that focus on the at-risk populations in the greatest need of intervention (i.e., those with the highest rates of obesity) [32]. This body of research would also benefit from an expansion of the target age range to include younger children (4–7 year-old), who are laying the foundation of their eating patterns [33], capable of participating in family meal preparations [34], and may reap benefits of family meals [35].

The primary objective of this study was to assess the impact of a 10-week multi-component family meals intervention study, Simple Suppers, tailored to racially diverse low-income children (4–10 year-old) and aimed at eliciting positive changes in child diet and health outcomes [36]. The hypothesis was that: 1) Diet (daily servings of fruit, vegetables, and sugar-sweetened beverages and Healthy Eating Index (HEI) score); z-scores for BMI, waist circumference, BP; weight status (healthy weight (5th to <85th percentile), overweight (≤85th percentile), obese (≤95th percentile); food preparation skills; and family meal outcomes (weekly frequency of dinner, breakfast, TV viewing during meals, and meals in a dining area) would improve more from baseline (T0) to post-intervention (T1) among children participating in the intervention compared to waitlist control participants. In addition, it was hypothesized that these outcomes would be maintained during the follow-up period (T1:T2) among intervention participants. Also, an exploratory analysis was conducted examining a potential dose-response effect factoring in the role of program attendance. Feasibility and acceptability were also assessed.

## Methods

### Study design

The Simple Suppers study was a quasi-experimental trial that targeted underserved racially diverse families with 4–10 year-old children [36]. The study was implemented over 12 months as a two-group trial using a staggered cohort design (Spring 2015 to Winter 2016). Each of the three cohorts was recruited across three time periods, separated by 10 weeks. Following baseline data collection (described below), families chose to enroll in either the immediate upcoming 10-week session of Simple Suppers (intervention group) or to wait for 10 weeks (waitlist control group), then begin the Simple Suppers program. Those in the waitlist control group did not receive any treatment during the waiting period. Randomization was not possible due to families’ personal schedules and desires to participate in the program with families they knew, as well as the need to build rapport with the community center and the families.

### Setting

The Simple Suppers intervention was implemented at a faith-based community center in Columbus, Ohio (Franklin County) that offers programming and services to those in need. Service area census tracts (year, 2016) demonstrated the following statistics in the center’s immediately surrounding neighborhoods: median household income was $32,307 to $58,490, compared to $51,890 in the broader county and $59,039 in the US; 10.7–24.9% of families falling below the poverty line, compared to 13.2% in the broader county and 12.7% in the US; 41.8% were Black compared to 21.2% in the county and 13.4% in the US; and a high percentage of households classified as families (58.7%).

### Participants

The study staff recruited families through center events, newsletter advertisements, and posters displayed in the center. To be eligible for study participation, caregivers had to be the primary food preparer in the home, responsible for at least one child 4–10 years old, speak English as the primary language in the home, and have lived in the US for at least one year. Families with one or more family members following a restrictive or therapeutic diet were excluded. For eligible families with multiple children 4–10 years old, all eligible children were invited to participate and complete measures.

### Data collection

Upon confirmation of study eligibility, a baseline data collection appointment was scheduled with the participating caregiver and child (ren) at the participant’s home or the community center during the two weeks preceding intervention commencement. Written caregiver consent, caregiver permission, and child assent were obtained prior to data collection. Data were collected via caregiver- completed questionnaires and direct measure at baseline (T0) and post-intervention (T1). At 10-week follow-up (T2), data were collected from intervention participants. A team of trained research staff (dietitians or undergraduate students majoring in nutrition, dietetics, or another health-related major (e.g., public health, nursing)), blinded from group assignment, facilitated data collection. Participating families received a $25 grocery store gift card at each data collection point. All study materials and procedures were approved by the Institutional Review Board at The Ohio State University.

### Intervention

The intervention design and delivery details are outlined elsewhere [36]. Briefly, Intervention Mapping, a protocol for developing theory- and evidence-based health promotion programs, was utilized in the development of the Simple Suppers intervention [37]. The Social Cognitive Theory, which posits that behavior change is a function of a reciprocal relationship between personal (e.g., behavioral capabilities, such as food preparation skills) and environmental (e.g., norms, modeling, and reinforcement) factors, served as the theoretical foundation for the Simple Suppers intervention [38]. Based on the current evidence linking family meals with improved child diet and weight status [16–19], program objectives for the Simple Suppers intervention were to: 1) ‘Increase frequency of family meals prepared in the home (≥5 days/week)’ and 2) ‘Improve child diet (significantly increase daily servings of fruits and vegetables to meet 2015 Dietary Guidelines recommendations; significantly decrease daily servings of sugar sweetened beverages; significantly increase HEI score).’

The Simple Suppers program included ten 90-min lessons delivered weekly over the dinner hour at the faith-based community center [36]. Each caregiver lesson topic focused on a barrier to family meals: making family mealtime fun; planning family meals on a budget; timesaving strategies for family meals; connecting with your child through family meals; planning well-balanced family meals; rethink your drink; making healthy cooking tasty and easy; serving and eating healthy portions; eating healthy when eating away from home; and planning fun and healthy snacks. Each child lesson focused on learning and practicing an age-appropriate food preparation skill. There were three session components built into each lesson. They included: an interactive group discussion and goal setting related to the weekly lesson topic with caregivers; hands-on food preparation activities with children (divided according to age group – 4-5 yr old; 6–8 yr old; 9–10 yr old); and group family meal with caregivers and children. The caregiver component was delivered by two trained program staff – a nutrition professional (e.g., dietitian, dietetic intern) and a community volunteer identified by the community center. The child component was delivered by a team of 5–10 trained program volunteers (e.g., dietetic interns, undergraduate students majoring in nutrition or dietetics, high school students).

### Study measures

At baseline (T0), caregivers completed a demographics questionnaire to assess child, caregiver, and household characteristics (age, sex, race (Black, White, or Other), income status, and food security) [39]. Low-income was categorically defined as participation in one or more of the following federal food assistance programs: Special Supplemental Nutrition Assistance Program for Women, Infants, and Children; Supplemental Nutrition Assistance Program; National School Lunch Program.

#### Dietary quality

Dietary intake was assessed by conducting three caregiver-assisted, nonconsecutive (two weekdays, one weekend day) 24-h dietary recalls using the United States Department of Agriculture’s 5-step multi-pass dietary recall method [40, 41]. At each data collection time point, the first was conducted during the in-person data collection visit, and the remaining two were conducted via telephone within two weeks of the initial in-person recall. The data were entered using the Nutrition Data System for Research, Version 2015 [42]. Typical daily dietary intake of intervention targets (fruit (servings/day), vegetables (servings/day), and sugar-sweetened beverages (servings/day)) was determined by averaging dietary intake across recalls at each time point. Diet quality was assessed by calculating an HEI 2010 total score [43].

#### Anthropometric and biometric assessments

Standardized procedures were used to measure children’s height (Hopkins Road Rod Portable Stadiometer) and weight (BalanceForm High Accuracy Digital Scale) and BMI calculated [44, 45]. Height and weight were measured twice and averaged. The Centers for Disease Control and Prevention (CDC) sex-specific BMI-for-age growth charts were used to calculate BMI percentiles and z-scores [44, 46]. Waist circumference was measured twice with a tape measure at the uppermost lateral border of the hip crest (ilium) and values averaged [44]. Child waist circumference z-scores were determined using CDC age- and sex-specific growth charts [47]. BP was measured three times via automated, calibrated BP monitors (Panasonic EW3109W) and a child blood pressure cuff; values were averaged. Age-, sex-, and height-adjusted National Heart Lung and Blood Institute charts were used to calculate systolic and diastolic BP z-scores [48].

#### Food preparation skills

Child food preparation skills were assessed with an 8-item questionnaire that was adapted from a previously validated questionnaire [22]. Three versions of the questionnaire were developed to align with the three program age groups, each demonstrating good internal consistency (4–5 yr old: α = 0.79; 6–8 yr old: α = 0.84; 9–10 yr old: α = 0.87). The stem statement was: “When we prepare food at home, my child is able to …” . Questions were situated on a 4-point scale (1 = Strongly Disagree (1 pt); 4 = Strongly Agree (4 pt)). For each item, there was a Don’t Know option (not scored). A sum score was calculated [1–32]. Specific questions according to age group are presented in Table 4.

#### Family meals

Frequency of family meals (dinner and breakfast), TV viewing during family meals, and eating family meals in a dining area were assessed with a modified questionnaire [22]. The stem statement was: “Over the past 7 days how many times …” . Questions were situated on a 5-point scale (0 = Never (0 pt), 1 = 1–2 times/week (1.5 pt), 2 = 3–4 times/week (3.5 pt), 3 = 5–6 times/week (5.5 pt), 4 = 7 times/week (7 pt). Scores were calculated for individual questions.

### Process measures

Feasibility (program dose and fidelity) and acceptability were assessed as process outcomes. To determine program feasibility, program dose was assessed by collecting weekly attendance (individual level). Program fidelity was determined by having a trained observer complete a program specific fidelity tool during each weekly lesson, which included a checklist of ‘yes’ or ‘no’ questions regarding key program components and activities, and program educators’ engagement with participants. At the end of the 10-week program, caregivers were asked to rate their child’s satisfaction of the program with a ‘yes’, ‘no’, or ‘unsure’ response to the question: “Did your child enjoy the Simple Suppers program?”

### Data analysis

All statistical analyses were conducted using Statistical Package for the Social Sciences software version 24 [49] with the exception of the mixed-effects ordinal regression model (described below) in which Statistical R software version 3.6.0 [50] was used. To evaluate intervention impact (hypothesis 1) at post-intervention (T1) data from each of the three cohorts were pooled and the intervention tested by comparing change (T1-T0) in dietary intake, anthropometric measures, BP, food preparation skills, and family meals from participants in the intervention compared to those in the waitlist control. Generalized linear mixed models were used to test for differences in the response variables of interest between groups (intervention and waitlist control) at post-intervention (T1). A mixed-effects ordinal regression model was used to test for differences in change in weight status category (coded categorically: 1) obese (≥95th percentile); 2) overweight (≥85th percentile); and 3) healthy weight (5th to <85th percentile) between groups at T1; note: children in the underweight (<5th percentile) category (*n* = 1 at T0 and *n* = 2 at T1) were collapsed into the healthy weight group). For dietary, anthropometric, BP, and food preparation skill variables, the potential confounders controlled for included: race (coded categorically: 1) Black, 2) White, 3) Other)); income (coded binomially: 1) low-income, 2) non low-income)); cohort; and baseline (T0) values of the response variables. Random effects were introduced for each family to account for expected correlations in outcomes among children within a single family. For the family meals variables, potential confounders controlled for included: race (coded categorically: 1) Black, 2) White, 3) Other)); income (coded binomially: 1) low-income, 2) non low-income)); cohort; caregiver race, sex, and age; oldest child race, sex, and age; baseline (T0) values of the response variables. To evaluate retention of change in outcomes among intervention participants (hypothesis 2), intervention group data from each of the three cohorts were pooled and change in dietary intake, anthropometric measures, BP, food preparation skills, and family meal outcomes among intervention participants at the end of the 10-week follow-up period was compared. Paired t-tests were used to assess change in outcomes of interest. For the exploratory dose analysis, intervention and waitlist control groups (assigned an attendance value of zero) were collapsed and GLMMs with attendance (continuous, 0–10), baseline values of responses, and potential confounders (outlined above) as independent variables were used.

For dietary outcomes, the protocol was to collect three dietary recalls at each time point. However, in many cases this was not feasible due primarily to challenges in contacting participants. In these instances, if two recalls were conducted, the average of the two recalls was used, and if a single 24-h dietary recall was collected, the single recall was used. Participant completion of 24-h dietary recalls at T0 was: 0 recalls (7%); 1 recall (68%); 2 recalls (12%); 3 recalls (13%). Participant completion of 24-h dietary recalls at T1 was: 0 recalls (39%); 1 recall (53%); 2 recalls (7%); 3 recalls (1%). Participant completion of 24-h dietary recalls at T2 was: 0 recalls (59%); 1 recall (37%); 2 recalls (4%); 3 recalls (0%).

Multiple imputations, calculated using Statistical R software version 3.6.0 [50], were used to deal with missing data with the exception of the dietary outcomes due to day-to-day variability in dietary intake. Imputation models were built with predicting variables including: cohort, income, sex, age, race, attendance, and group assignment as well as dependent variables of interest when imputing an independent variable for analysis models. Fifty imputation iterations were run for each missing value and convergence was met. Statistical significance was established at *p* < 0.05.

## Results

**Fig. 1:**
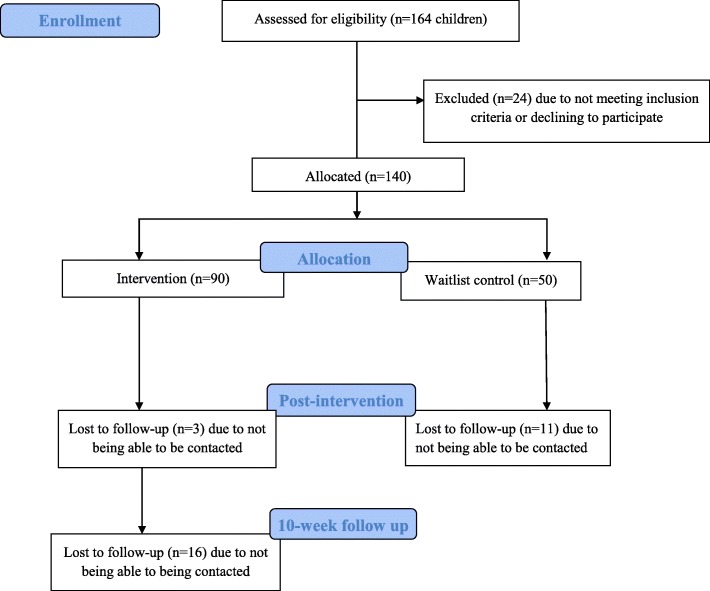
CONSORT Flow Diagram of the 10-Week Quasi-Experimentally Designed Simple Suppers Study for 4- to 10-Year Old Children

One-hundred and forty children enrolled (109 families) and 126 children completed T1 (90% participant retention; Fig. 1). Seventy-one of 87 intervention participants who completed the T1 assessment completed T2 (81.6% retention). Descriptive summaries of participant baseline characteristics are presented in Table 1. Characteristics did not differ by group assignment (intervention and waitlist control) with the exception of race which was controlled for in subsequent analyses.

**Table 1:** Baseline (T0) Demographics of 126 4- to 10-Year-Old Children and their Household Characteristics by Intervention and Waitlist Control Group Assignment^a,b^

Child Characteristics	Total (*n* = 126)	Intervention (*n* = 87)	Waitlist Control (*n* = 39)	*P*-value
Age (years)^c^, mean (SD)	6.9 (1.9)	6.8 (1.9)	7.2 (2.1)	0.24
Sex (female)^d^, n (%)	76 (60)	52 (60)	24 (62)	0.91
Race^d^, n (%)				
Black	73 (60)	53 (63)	20 (51)	**0.007**
White	32 (25)	15 (17)	17 (41)	
Other^e^	18 (15)	16 (20)	2 (8)	
Anthropometrics and Biometrics^c^, mean (SD)				
Body Mass Index z-score^f^	0.64 (1.2)	0.64 (1.2)	0.66 (1.3)	0.93
Waist Circumference z-score^f^	0.71 (1.0)	0.71 (1.0)	0.69 (1.2)	0.90
Systolic Blood Pressure z-score^f^	1.60 (1.3)	1.60 (1.3)	1.49 (1.2)	0.53
Diastolic Blood Pressure z-score^f^	1.23 (1.3)	1.23 (1.2)	1.06 (1.2)	0.33
Household Characteristics	Total (*n* = 98)	Intervention (*n* = 66)	Waitlist Control (*n* = 32)	*P*-value
Household Income Status^d,g^, n (%)				
Low-Income	36 (40)	24 (39)	12 (41)	0.49
Non Low-Income	55 (60)	38 (61)	17 (59)	
Home Food Security^d,h^, n (%)				
High/Marginal Food Security	60 (63%)	35 (56%)	25 (78%)	0.08
Low Food Security	19 (20%)	16 (25%)	3 (9%)	
Very Low Food Security	16 (17%)	12 (19%)	4 (13%)	

^a^Numbers presented do not represent imputed values. There were missing data for: age (*n* = 2); sex (n = 1); race (n = 3); anthropometrics (*n* = 10); income (n = 7); and food security (*n* = 3).^b^Participant response rates varied therefore sample sizes are provided for each outcome variable

^c^One-Way ANOVA for continuous variables

^d^Chi-square for categorical variables

^e^Includes participants who identified as Alaska Native/American Indian, Asian, Native Hawaiian/Pacific Islander, or Mixed Race

^f^z-score of 0 = 50% percentile, 1 = 84% percentile, 2 = 97.5% percentile

^g^Low-income defined as participation in one or more of the following federal food assistance programs: Special Supplemental Nutrition Assistance Program for Women, Infants, and Children (WIC); Supplemental Nutrition Assistance Program (SNAP); National School Lunch Program (NSLP)

^h^USDA 6-item Short Form Home Food Security Questionnaire. A score of 0–1 = High/marginal food security, 2–4 = Low food security; 5–6 = Very low food security

All p-values that are signicant (<0.05) are bolded.

Intervention versus waitlist control children demonstrated significantly higher food preparation skills (*p* < 0.001; CI: 2.88, 6.58) and lower TV viewing during family meals (*p* = 0.04; CI: − 2.06, − 0.02) at post-intervention (T1) (Table 2). No intervention effects were found on dietary intake or BP outcomes. Regarding weight status outcomes, there were no intervention effects on BMI or waist circumference z-scores, however intervention versus waitlist controls experienced a greater change toward healthy weight (p = 0.04). The increase in food preparation skills among intervention children (T0:T1) was maintained at the 10-week follow-up (T2) (Table 3). An examination of individual food preparation skills among intervention participants during the intervention period (T0:T1) according to age group (4–5 yr old; 6–8 yr old; 9–10 yr old) demonstrated that the increases were significant only for children in the youngest age categories (4–5 yr old (set dinner table, peel soft foods, measure dry ingredients, measure liquid ingredients, cut soft foods, grease/spray pan); 6–8 yr old (set dinner table, pour beverages, measure dry ingredients, measure liquid ingredients, crack eggs, grate cheese, cut soft foods, wash hands); however, when examining change throughout the entire study period (i.e., baseline (T0) to follow-up (T2)), significant increases among the older age group (9–10 yr old) emerged (core/slice apple, grate cheese, peel potato/other vegetable, and use food thermometer) (Table 4).

**Table 2:** By-Group (Intervention vs. Waitlist Control Child Participants, ages 4- to 10-Years Old) Effects of the 10-Week Simple Suppers Intervention: Difference at Post-Intervention (T1) Controlling for Baseline (T0) Values

Outcomes	Intervention			Waitlist Control			β	*P*-Value	95% CI
	T0	T1	Δ	T0	T1	Δ			
Dietary Intake^a,b,c^									
Total Fruit^d^ (servings/day)	1.68 (1.2)	1.71 (1.8)	0.04 (2.1)	1.86 (1.8)	1.48 (1.2)	-0.38 (1.9)	0.42	0.38	(-0.83, 2.11)
Whole Fruit (servings/day)	1.15 (0.9)	1.33 (1.7)	0.18 (1.8)	1.39 (1.5)	1.12 (1.2)	-0.27 (1.7)	0.45	0.15	(-0.40, 2.42)
Total Vegetable (servings/day)	1.69 (1.4)	2.05 (1.6)	0.36 (1.4)	1.70 (1.6)	1.60 (1.7)	-0.10 (2.8)	0.46	0.41	(-0.76, 1.84)
Sugar-Sweetened Beverage (servings/day)	0.41 (0.6)	0.39 (0.7)	-0.02 (0.9)	0.55 (0.8)	0.48 (0.7)	-0.07 (0.8)	0.05	0.14	(-0.93, 0.14)
Healthy Eating Index Total Score (0-100)	52.24 (10.5)	53.21 (11.6)	0.97 (16.1)	53.25 (11.4)	55.81 (9.4)	2.56 (14.6)	-1.59	0.86	(-9.37, 11.12)
Anthropometrics and Biometrics									
Body Mass Index z-score^e^	0.68 (1.1)	0.52 (1.2)	-0.16 (0.6)	0.74 (1.3)	0.68 (1.4)	-0.06 (0.3)	-0.10	0.26	(-0.39, 0.11)
Weight Status^f,g^, n (%)									
Underweight	1 (1.1)	2 (2.3)	1 (1.1)	0 (0.0)	0 (0.0)	0 (0.0)	17.2	**0.04**	(1.1, 262.2)
Normal Weight	56 (64.4)	63 (72.4)	7 (8.0)	25 (64.1)	24 (61.5)	-1 (2.6)			
Overweight	17 (19.5)	10 (11.5)	-7 (8.0)	3 (7.7)	4 (10.3)	1 (2.6)			
Obese	13 (14.9)	12 (13.8)	-1 (1.1)	11 (28.2)	11 (28.2)	0 (0.0)			
Waist Circumference z-score^e^	0.76 (0.9)	0.79 (0.9)	0.03 (0.6)	0.71 (1.3)	0.71 (1.2)	0.00 (1.2)	0.03	0.10	(-0.39, 0.06)
Systolic Blood Pressure z-score^e^	1.41 (1.3)	1.27 (1.4)	-0.14 (1.6)	1.55 (1.1)	1.71 (1.6)	0.16 (1.9)	-0.30	0.21	(-0.89, 0.20)
Diastolic Blood Pressure z-score^e^	1.31 (1.4)	1.09 (1.4)	-0.22 (1.9)	1.10 (1.3)	1.39 (1.8)	0.29 (2.1)	-0.51	0.88	(-0.91, 0.78)
Food Preparation Skill Ability^c,h^, mean (SD)	22.03 (5.1)	26.89 (4.6)	4.86 (5.1)	23.21 (4.8)	22.85 (5.5)	-0.36 (4.2)	5.22	**<0.001**	(2.88, 6.58)
Family Meals (meals/week)^i^, mean (SD)									
Family dinner frequency	4.77 (2.1)	4.42 (2.1)	-0.35 (2.8)	5.17 (1.5)	4.02 (2.0)	-1.15 (2.4)	0.80	0.39	(-0.63, 1.59)
Family breakfast frequency	2.66 (2.3)	2.75 (2.4)	0.09 (3.0)	2.73 (2.3)	2.80 (2.4)	0.07 (3.4)	0.16	0.98	(-1.25, 1.23)
Family meal with TV	1.93 (1.9)	2.15 (2.2)	0.22 (2.4)	2.50 (2.2)	2.94 (2.5)	0.44 (2.2)	-0.22	**0.04**	(-2.06, -0.02)
Family meals in a dining area	4.09 (2.4)	4.31 (2.2)	0.22 (2.4)	3.91 (2.6)	4.35 (2.5)	0.44 (2.2)	-0.12	0.82	(-1.31, 1.05)

^a^Daily intake averaged across caregiver-assisted, nonconsecutive 24-hr dietary recalls collected using USDA five-step multiple-pass method

^b^Participant completion of 24-hr dietary recall at T0: 0 recalls (7%); 1 recall (68%); 2 recalls (12%); 3 recalls (13%). Participant completion of 24-hr dietary recall at T1: 0 recalls (39%); 1 recall (58%); 2 recalls (4%); 3 recalls (0%)

^c^By-group differences at T1 determined by generalized linear mixed modeling controlling for: cohort; household income; child race, sex, age; group assignment; participant id (family id); baseline value of outcome variable (Y (outcome variable)= outcome at T1)

^d^Total fruit= whole fruit + 100% fruit juice

^e^By-group differences at T1 determined by generalized linear mixed modeling controlling for: cohort; household income; child race; group assignment; participant id (family id); baseline value of outcome variable (Y (outcome variable)= outcome at T1)

^f^Mixed-effects ordinal regression model to test for differences in change in weight status category (1=obese (≥95^th^ percentile), 2=overweight (≥85^th^percentile), and 3=healthy weight (5^th^ to <85^th^ percentile)) between groups at T1; note: children in the underweight (<5^th^ percentile) category (n=1 at T0 and n=2 at T1) were collapsed into the healthy weight group)

^g^Odds ratio (OR) and corresponding OR confidence intervals presented

^h^The stem statement was: “When we prepare food at home, my child is able to…”. Questions were situated on a 4-point scale (1=Strongly Disagree (1pt); 4=Strongly Agree (4pt)). A sum score was calculated (1-32)

^i^By-group differences determined by generalized linear modeling controlling for: cohort; household income; caregiver race, sex, and age; oldest child race, sex, and age; baseline value; group assignment (Y (outcome variable)= outcome at T1)

All p-values that are signicant (<0.05) are bolded.

**Table 3:** Within Group Differences among Simple Suppers Intervention Children Participants (4- to 10-Years Old) in Outcomes at Baseline (T0), Post-Intervention (T1), and Follow-up (T2)

Outcomes	T0	Δ T0 to T1	P-value^e^	Δ T1 to T2	P-value^e^	Δ T0 to T2	*P*-value^e^
Dietary Intake^a,b^, mean (SD)							
Total Fruit^c^ (servings/day)	1.68 (1.2)	0.03 (2.1)	0.91	0.13 (1.6)	0.28	0.16 (1.4)	0.32
Whole Fruit (servings/day)	1.15 (0.9)	0.18 (1.8)	0.43	−0.17 (1.6)	0.61	0.01 (1.1)	0.70
Total Vegetable (servings/day)	1.69 (1.4)	0.36 (1.4)	0.09	−0.37 (1.4)	0.71	−0.01 (1.3)	0.67
Sugar-Sweetened Beverages (servings/day)	0.41 (0.6)	−0.02 (0.9)	0.88	0.00 (1.0)	0.89	−0.02 (0.8)	0.80
Healthy Eating Index Total Score (0–100)	52.24 (10.5)	0.97 (16.1)	0.21	1.90 (10.8)	0.27	2.87 (10.1)	0.13
Anthropometrics and Biometrics, mean (SD)							
Body Mass Index z-score	0.68 (1.1)	−0.16 (0.6)	**0.03**	0.06 (1.1)	0.36	−0.10 (0.9)	0.13
Waist circumference z-score	0.76 (0.9)	0.03 (0.6)	0.66	−0.15 (0.9)	0.06	−0.12 (0.7)	0.16
Systolic Blood Pressure z-score	1.41 (1.3)	−0.14 (1.6)	0.50	−0.51 (1.6)	**0.04**	−0.66 (1.3)	**0.01**
Diastolic Blood Pressure z-score	1.31 (1.4)	−0.22 (1.9)	0.27	−0.27 (1.2)	0.45	−0.49 (1.5)	**0.01**
Food Preparation Skill Ability^d^, mean (SD)	22.03 (5.1)	4.86 (5.1)	**< 0.001**	0.04 (4.6)	0.93	4.90 (4.8)	**< 0.001**
Family Meals (meals/week), mean (SD)							
Family dinner frequency	4.77 (2.1)	−0.35 (2.8)	0.26	0.19 (2.0)	0.51	−0.16 (2.1)	0.56
Family breakfast frequency	2.66 (2.3)	0.09 (3.0)	0.66	1.08 (2.4)	**0.007**	1.24 (2.2)	**0.001**
Family meal with TV	1.93 (1.9)	0.22 (2.4)	0.59	0.03 (1.9)	0.72	0.20 (1.9)	0.71
Family meals in a dining area	4.09 (2.4)	0.22 (2.4)	0.39	0.33 (2.0)	0.32	0.59 (1.8)	**0.04**

^a^Daily intake averaged across caregiver-assisted, nonconsecutive 24-h dietary recalls collected using USDA five-step multiple-pass method

^b^Participant completion of 24-h dietary recalls at T0: 0 recalls (7%); 1 recall (68%); 2 recalls (12%); 3 recalls (13%). Participant completion of 24-h dietary recalls at T1: 0 recalls (39%); 1 recall (53%); 2 recalls (7%); 3 recalls (1%). Participant completion of 24-h dietary recalls at T2: 0 recalls (59%); 1 recall (37%); 2 recalls (4%); 3 recalls (0%)

^c^Total fruit = whole fruit + 100% fruit juice

^d^The stem statement was: “When we prepare food at home, my child is able to …”. Questions were situated on a 4-point scale (1 = Strongly Disagree (1 pt); 4 = Strongly Agree (4 pt)). A sum score was calculated (1–32)

^e^Within-group differences examined using paired t-test

All p-values that are signicant (<0.05) are bolded.

**Table 4:** Within Group Differences among Simple Suppers Intervention Child Participants (4- to 10-Years Old) in Food Preparation Skills according to Age Group at Baseline (T0), Post-Intervention (T1), and Follow-Up (T2)

Food Preparation Skills	T0	Δ T0 to T1	*P*-value^b^	Δ T1 to T2	*P*-value^b^	Δ T0 to T2	*P*-value^b^
4–5 year old^a^, mean (SD)							
Set dinner table	2.93 (1.0)	0.71 (0.6)	**0.04**	0.07 (0.8)	0.34	0.78 (0.9)	**0.04**
Wipe table	3.21 (0.6)	−0.15 (0.7)	0.58	0.17 (0.5)	0.50	0.32 (0.7)	0.30
Peel soft foods	3.14 (0.8)	0.57 (0.8)	**0.01**	0.05 (0.6)	0.59	0.62 (0.6)	**0.02**
Measure dry ingredients	2.29 (0.9)	1.00 (0.7)	**0.01**	−0.05 (0.7)	0.79	0.95 (0.9)	**< 0.01**
Measure liquid ingredients	2.31 (0.9)	0.92 (0.7)	**0.02**	0.24 (0.8)	0.19	1.16 (0.7)	**< 0.001**
Cut soft foods	2.54 (1.0)	0.84 (1.1)	**0.01**	0.09 (0.6)	0.99	0.93 (0.6)	**0.02**
Grease or spray pan	2.43 (1.0)	0.71 (0.9)	**0.03**	0.68 (0.7)	**0.01**	1.39 (0.6)	**< 0.001**
Wash hands	3.14 (0.7)	0.22 (0.6)	0.57	0.17 (1.0)	0.52	0.39 (0.8)	**0.02**
6–8 year old^a^, mean (SD)							
Set dinner table	2.82 (1.1)	0.64 (0.9)	**0.04**	0.30 (0.8)	0.10	0.94 (1.0)	**0.04**
Pour beverages	3.30 (0.7)	0.59 (0.4)	**< 0.001**	−0.10 (0.3)	0.67	0.49 (0.5)	**0.02**
Measure dry ingredients	2.68 (1.0)	0.84 (1.1)	**< 0.01**	−0.04 (0.5)	0.26	0.88 (0.7)	**0.01**
Measure liquid foods	2.68 (1.0)	0.92 (0.8)	**< 0.001**	−0.04 (0.7)	0.72	0.88 (0.9)	**< 0.001**
Crack eggs	2.64 (1.0)	1.00 (0.9)	**< 0.001**	−0.16 (0.4)	0.75	0.84 (0.6)	**< 0.001**
Grate cheese	2.14 (1.2)	1.05 (1.0)	**< 0.01**	0.06 (0.8)	0.06	1.11 (1.0)	**< 0.001**
Cut soft foods	2.96 (1.0)	0.65 (0.8)	**< 0.001**	0.11 (0.4)	0.67	0.76 (0.9)	**< 0.01**
Wash hands	3.21 (0.7)	0.66 (0.6)	**< 0.001**	−0.23 (0.6)	0.51	0.43 (0.6)	0.58
9–10 year old^a^, mean (SD)							
Follow a recipe	2.33 (1.4)	0.67 (1.1)	0.14	−0.09 (1.0)	0.99	0.58 (1.0)	0.39
Crack eggs	3.44 (0.7)	0.12 (0.5)	0.59	−0.06 (0.6)	0.98	0.06 (0.5)	0.34
Core and slice apple	2.22 (1.0)	0.88 (0.8)	0.12	0.09 (0.6)	0.62	0.98 (0.7)	**0.05**
Grate cheese	2.38 (1.1)	0.87 (0.9)	0.06	−0.03 (0.8)	0.62	0.84 (0.9)	**0.02**
Peel potatoes/other vegetable	2.33 (1.0)	0.89 (0.6)	0.07	−0.02 (0.9)	0.99	0.87 (0.8)	**0.05**
Cut chicken w/ knife/scissors	2.75 (0.9)	0.50 (0.8)	0.23	−0.52 (0.7)	0.37	−0.02 (0.8)	0.34
Use a food thermometer	2.29 (1.1)	1.14 (0.5)	0.07	−0.43 (0.8)	0.98	0.71 (1.0)	**0.02**
Wash hands	3.11 (0.6)	0.45 (0.6)	0.10	−0.01 (0.4)	0.36	0.44 (0.7)	0.68

^a^The stem statement was: “When we prepare food at home, my child is able to …”. Questions were situated on a 4-point scale (1 = Strongly Disagree (1 pt); 4 = Strongly Agree (4 pt)). A sum score was calculated (1–4)

^b^Differences examined using paired t-test

All p-values that are signicant (<0.05) are bolded.

In the exploratory dose analysis, attendance level was inversely related to post-intervention (T1) BMI z-score (*p* = 0.02; CI: − 0.07, − 0.01), systolic BP z-score (*p* = 0.03; CI: − 0.14, − 0.01), and TV viewing during meals (*p* = 0.02; CI: − 0.26, − 0.02), and positively related to food preparation skills (*p* < 0.001; CI: 0.23, 0.71) (Table 5).

**Table 5:** Dose-Effect Analysis of the 10-Week Simple Suppers Intervention among Intervention and Waitlist Control Group Children (4- to 10-Years Old): Difference at Post-Intervention (T1)^a^

	β (SE)	*P*-value	95% CI
Dietary Intake^b,c,d^			
Total Fruit^e^ (servings/day)	0.07 (0.1)	0.42	(-0.10, 0.24)
Whole Fruit (servings/day)	0.11 (0.1)	0.19	(-0.06, 0.27)
Total Vegetable (servings/day)	0.05 (0.1)	0.53	(-0.10, 0.20)
Sugar-Sweetened Beverages (servings/day)	-0.03 (0.1)	0.33	(-0.09, 0.03)
Healthy Eating Index Total Score (0-100)	0.01 (0.6)	0.99	(-1.15, 1.13)
Anthropometrics and Biometrics^d^			
Body Mass Index z-score	-0.04 (0.0)	**0.02**	(-0.07, -0.01)
Waist Circumference z-score	-0.02 (0.0)	0.32	(-0.05, 0.02)
Systolic Blood Pressure z-score	-0.07 (0.0)	**0.03**	(-0.14, -0.01)
Diastolic Blood Pressure z-score	-0.01 (0.1)	0.85	(-0.09, 0.10)
Food Preparation Skill Ability^f,d^	0.47 (0.1)	**<0.001**	(0.23, 0.71)
Family Meals (meals/week)^g^			
Family dinner frequency	0.04 (1.1)	0.49	(-0.09, 0.17)
Family breakfast frequency	-0.01 (0.1)	0.85	(-0.16, 0.13)
Family meal with TV	-0.14 (0.1)	**0.02**	(-0.26, -0.02)
Family meals in a dining area	-0.03 (0.1)	0.58	(-0.17, 0.10)

^a^Includes all study participants (intervention and waitlist controls). Participants in the waitlist control group were assigned an attendance level of ‘0’

^b^Daily intake averaged across caregiver-assisted, nonconsecutive 24-hr dietary recall collected using USDA five-step multiple-pass method

^c^Participant completion of 24-hr dietary recalls at T0: 0 recalls (7%); 1 recall (68%); 2 recalls (12%); 3 recalls (13%). Participant completion of 24-hr dietary recalls at T1: 0 recalls (39%); 1 recall (58%); 2 recalls (4%); 3 recalls (0%)

^d^Dose-effect (attendance as continuous variable, 0-10 (lessons)) determined by generalized linear mixed modeling controlling for: cohort; household income; child race, sex, age; participant id (family id); baseline value of outcome variable (Y (outcome variable)= outcome at T1)

^e^Total fruit=whole fruit + 100% fruit juice

^f^The stem statement was: “When we prepare food at home, my child is able to…”. Questions were situated on a 4-point scale (1=Strongly Disagree (1pt); 4=Strongly Agree (4pt)). A sum score was calculated (1-32)

^g^Dose-effect (attendance as continuous variable, 0-10 (lessons)) determined by generalized linear mixed modeling controlling for: cohort; household income; caregiver race, sex, and age; oldest child race, sex, and age; baseline value of outcome variable (Y (outcome variable)= outcome at T1)

All p-values that are signicant (<0.05) are bolded.

Program dose, defined as mean (SD) attendance rate, was 6.98 (2.3) sessions or 70% of the sessions. The majority of children (68%, *n* = 55) attended 7–10 lessons, 28% (*n* = 24) attended 4–6 lessons, and 9% (*n* = 8) attended 1–3 lessons. Approximately 95% of lessons were delivered as intended and child participants were engaged in the program activities 96% of the time. At program completion (T1), 100% of participating caregivers reported their child (ren) enjoyed participating in the program.

## Discussion

The present study assessed the impact of a 10-week, multi-component family meals intervention study aimed at eliciting positive changes in child diet, weight status, and health among 4–10-year-old children from racially diverse families from an underserved community. It was hypothesized that diet, z-scores for BMI, waist circumference, and BP, weight status, food preparation skills, and family meal outcomes would improve more from baseline to post-intervention among children in the intervention versus waitlist control group. Results demonstrated an intervention impact on food preparation skills and certain family mealtime routines (i.e., TV viewing), but not diet or z-scores for BMI, waist circumference, or BP. However there was an impact on weight status such that intervention versus waitlist controls experienced a greater change toward healthy weight. This finding parallels results from the Korea National Health and Nutrition Examination Survey or KNHANES in which the odds of obesity was inversely associated with family meals among 2904 elementary-aged children [20], and indicate a true preventive effect of healthy family mealtime routines among children at greatest risk for obesity (i.e., racial minority children, particularly those from low-income households).

With regards to the intervention impact on food preparation skills, results parallel findings from the HOME randomized controlled trial, a childhood obesity prevention intervention aimed at increasing family meals among 8–10 year-old children, in which intervention versus control children demonstrated higher food preparation skills at post-intervention [22]. In addition, though the setting was different from the current study (school vs faith-based center), results parallel findings from a narrative review of culinary interventions for children (5 to 12 years old) in which consistent positive impacts on cooking skills were observed [51]. Given emerging evidence associating food preparation skills with better long-term health [52], this warrants further investigation. However, there is need for caution given that food preparation skills may not always guarantee a healthy diet [53–55].

Among the family meal outcomes (weekly frequency of family meals (dinner and breakfast), TV viewing during meals, and having a family meal in a dining area), decreased TV viewing was the only consistent finding. Specifically, there was an intervention impact on TV viewing during meals (intervention participants), as well as a dose effect on TV viewing (intervention and waitlist control participants). The majority of evidence demonstrates an inverse association between TV viewing during meals and child diet quality [56]. On the other hand, the data demonstrating an association between TV during and child weight status are mixed demonstrating negative [57] or null [58, 59] effects. The mechanism is unclear, though may be due to decreased distractions and resulting improvements in family dynamics and sense of connectedness [59]. The absence of an intervention effect on family meal frequency goes against the study hypothesis, but may not be important as the number of times a family reports having a meal together doesn’t necessarily reflect the quality of the meal. It will be critical for future work in this line of inquiry to expand assessment methods to include a tool to assess the healthfulness of meals served to children [60].

The lack of an intervention impact on dietary targets (daily servings of fruit, vegetables, and sugar-sweetened beverages, and diet quality or HEI), contradicts the majority of evidence and [16–19, 22] the theoretical underpinnings of the Simple Suppers intervention (i.e., Social Cognitive theory which purports that skills lead to behavior change) [36, 38]. However, it is possible that child dietary intake improvements did in fact occur, but changes were not detected given the low response rate for the 24-h dietary recall by caregivers. The low response rate, particularly at post-intervention (T1) and follow-up (T2), was due primarily to difficulty contacting participants (e.g., changed cellular service and number, changed address). This is not an uncommon barrier in working with economically disadvantaged populations, however may be overcome by greater reliance on the community partner with regards to contacting and monitoring participants [61] and therefore should be explored in future work. The low response rate might also have been due to the burdensome nature of dietary recalls; this is a common problem in collecting these data and therefore important for future efforts to identify ways to overcome this issue through alternative validated approaches that are less arduous and similarly accurate in terms of estimating intake (e.g., digital assessment through smartphone technology) [62–64].

The lack of an intervention effect on BMI z-score finding is similar to results from the HOME [22] and HOME Plus randomized controlled trial [24] in which there were no significant treatment group differences in BMI z-scores at post-intervention. However, results from the HOME Plus trial did demonstrate that when accounting for pubertal status, prepubescent intervention children had significantly lower BMI z-scores vs prepubescent control group children. These findings suggest that younger children may be more responsive to a weight status change resulting from healthy family mealtime routines; however, this hypothesis does not align with findings from the Simple Suppers study which primarily included prepubescent children (4–10 year-old). Regardless, it will be important for future work in this line of inquiry to examine potential BMI z-score responders (e.g., prepubescent vs pubescent) in reference to healthy family mealtime routines.

A second hypothesis in the current study was that improvements in dietary intake and quality and z-scores for BMI, waist circumference, BP, food preparation skills, and family meals outcomes would be maintained during the follow-up period among intervention participants. A retention of food preparation skills at the 10-week follow up was demonstrated. The lasting change in food preparation skills is a powerful finding and may be explained by utilization of the intervention mapping protocol (i.e., selection of theoretical methods and practical strategies targeted to determinants of behavior) in the design of Simple Suppers [36, 37]. Specifically, this Social Cognitive Theory-based intervention was designed to build behavioral capabilities (i.e., skills) in food preparation by teaching children age-appropriate food preparation skills at each lesson (facilitation), having children learn these skills by being in groups of children their own age (vicarious learning), and practicing the skills at each weekly lesson and at home (mastery experience).

A further examination of individual food preparation skills among intervention participants according to age group (4–5 yr old; 6–8 yr old; 9–10 yr old) during the intervention period demonstrated that the increases were significant only for children in the youngest age categories (4–5 yr old; 6–8 yr old). These data support that young children are indeed capable of participating in meal preparation [65], and is significant in part because they are laying the foundation for future eating patterns [33]. It was also observed that there were significant increases among the older age group (9–10 yr old) over the entire study period, from baseline to the 10-week follow-up. It is possible that because the skills were more advanced, even age adjusted, it took more time to master them [38].

The exploratory dose analysis factoring in attendance level demonstrated that each additional lesson was associated with a lower BMI z-score. Importantly, results remained similar in terms of directionality and significance when examining intervention participants alone (data not shown). This finding bears similarity to results from some observational studies [16–20] and experimental trials demonstrating a positive impact of family or shared meals on child weight status [10–12, 22], thereby strengthening the evidence-base in favor of American Academy of Pediatrics’ recommendation to engage in healthy family mealtime routines as a childhood obesity prevention strategy [6]. On the other hand, it is possible that a selection bias occurred whereby those who attended more versus less were different in some unaccounted way (e.g., higher motivation, fewer logistic barriers such as transportation) [66]. It is also possible that the observed positive dose-response was due to increased contact and not the intervention itself. This possibility cannot be ruled out given that the study design did not include an attention control group [67].

One of the novel aspects of the current study was inclusion of BP. Hypertension in childhood is associated with obesity and early onset diabetes [24] Given the American Academy of Pediatrics’ recommendation to engage in family meals as an obesity prevention strategy, there is need to expand the understanding of the relationship between family meals and child weight status to critical biomarkers of health like BP. In the exploratory analysis, we demonstrated an inverse association between attendance level and systolic BP z-score. These findings are consistent with results from a cross-sectional study of 723 US adolescents residing in a Midwest state demonstrating associations of family dinner from fast-food restaurants and take-out sources with increased metabolic risk cluster z-scores (which included systolic BP) [68]. These findings also bear similarity to results from a 12-week gardening, nutrition, and cooking intervention in primarily Latino elementary-age children (*n* = 70 control and *n* = 34 intervention) whereby children in the intervention group had decreased diastolic BP [69]. Worth noting, there is strong evidence in favor of systolic versus diastolic BP being predictive of mortality [70]. Regardless, these data taken together point to the need to further explore the relationship of family mealtime routines and child BP.

Another novel feature of the current study was the delivery setting – i.e., faith-based community center – as few childhood obesity interventions have occurred in this space [25, 71]. Among the limited number of studies, results demonstrate that faith-based community centers are highly effective in engaging families in programming [71, 72], particularly for the African American community in which currently and historically the church is viewed as central to a community and daily living [73]. This may at least partially explain the strong feasibility (70% attendance), acceptability (100% satisfaction), and retention (87.5%) results. It is also possible that children’s enjoyment of the program spurred engagement. Specifically, based on the caregiver-completed child satisfaction survey, and a small sample of post-intervention exit interviews (*n* = 4; data not shown), children reported that what they enjoyed most about the program was preparing recipes (“*oh, I liked when we made dessert – the yogurt parfaits” [Black female, 7 yr] and “um … they [the recipes] weren’t hard to make!” [Hispanic White female, 8 yr]*. In the future, it will be important to understand the main contributing factors for the observed high feasibility and acceptability to assure successful scale up.

It will also be important to examine ways to further minimize programmatic costs in future scale-up studies. In the current study, $25 per family was budgeted for weekly programmatic expenses. While this amount ($25) is the same or less than as what it would cost for a family to eat out at a fast food or fast casual restaurant [74] and this expense decreased throughout the study (data not shown) due to improvements in the program staff’s ability to ‘grocery shop on a budget’, additional cost savings strategies such as assessing a nominal fee to participating families should be explored.

The present study had multiple strengths. First, the Simple Suppers intervention was designed utilizing the intervention mapping protocol, peer-reviewed by a team of experts in the field, and pilot tested for feasibility [36]. In addition, outcomes were assessed using reliable questionnaires and established protocols by trained researchers. In addition, this study was the first family meals intervention to expand health outcomes beyond weight status (i.e., BP). The setting of a faith-based community center was also novel, and facilitated engagement in programming. Additionally, the study population was broader than the populations in previous family meals research. The current study included greater racial and socioeconomic diversity, allowing for increased generalizability to minority and at-risk groups. The inclusion of all children in the family who met age criteria was also unique to the current study.

A major limitation of the current study was lack of randomization and thus potential introduction of selection bias. That said, between-group differences were assessed at baseline and differences (child race) were accounted for in all statistical models to help minimize any bias that may have been introduced. Recruiting at various (multiple) times throughout the year helped to reduce the chance of bias because much of the programming at the center was seasonal and therefore there was a regular influx of new families to the center (e.g., basketball in winter, soccer in spring). Regardless, the difficulty with randomization, inherent to community-based research, needs to be better understood and factored into future research [75]. In addition, the study was designed for low-income family households; identification of this specific target population was assured by recruiting from a community center whose mission is to serve families in economic and social need and is situated in a part of the City surrounded by this target population. However, data from the current study demonstrated recruitment of a mixed income population. That said, the measure of income status has imprecisions (i.e., reliance on federal food assistance programs). It will be important for future work to include a more sensitive or comprehensive assessment of income status [76]. Use of a self-report dietary assessment method (i.e., 24-h diet recall) represents another study limitation due to the well-documented issues of subject recording bias, subject selection bias, and motivation [77]. Additional issues arise in assessing diet in children, particularly those between the ages 4 and 10 years old, due to issues of literacy and writing skills, limited skills in recognizing foods, memory constraints, difficulty conceptualizing time, limited attention span, and inability of parents to give a detailed assessment of what their children eat when away from home or in the care of others [77]. There is consensus that the caregiver-assisted 24-h multiple pass recall conducted over at least a 3-day period (weekdays and weekend days) is the best method for overcoming these issues, and accurately estimating dietary intake for children in this specific age group (4 to 10 or 11 years old) [40]. Importantly, the current study relied on this methodology. However, in many cases only a single dietary recall was collected due to difficulty contacting participants, ultimately reducing the ability to capture a ‘picture’ reflective of usual or typical intake. A separate but related issue was the relatively high proportion of participants with no dietary recalls at post-test (T1) or follow-up (T2), also due to difficulty contacting participants. It is possible though not likely that this introduced a nonresponse bias as precautions were taken to protect against high respondent burden, judgmental comments by interviewers, or language/cultural barriers (i.e., extensive piloting and staff training) [78]. Regardless, it will be critical for future research in this line of inquiry (i.e, family meals interventions in underserved racially diverse children and caregivers occurring in the faith-based community setting) to develop strategies for overcoming barriers to achieving complete dietary data collection. A final limitation was the use of only one closed-ended question to assess program acceptability. A forced-choice response may have made participants feel obligated to answer “yes” due to social desirability bias or to please researchers. Future interventions would benefit from expanding the assessment of program acceptability via in-depth interviews to better understand what participants did or did not enjoy and allow them to expand upon their answers.

## Conclusion

High feasibility of the Simple Suppers study was demonstrated, as well as improvements in children’s weight status, food preparation skills, and TV viewing during meals among intervention versus control participants, although no overall impacts on diet or z-scores for BMI, waist circumference, or BP. Increased food preparation skills and decreased TV viewing during meals were retained among intervention participants at study follow-up, and change in food preparation skills throughout the study period varied by age group. In addition, a dose response was observed on z-scores for BMI and systolic BP, as well as TV viewing during meals, indicating that the program did have an impact among participants who attended more versus less. To our knowledge, this is the first study of its kind in that it included racial and income diverse children and families. Collectively, findings from the current study indicate the importance of targeting populations at increased risk for childhood obesity.

## Data Availability

The datasets used and/or analysed during the current study are available from the corresponding author on reasonable request.

## Abbreviations

BMI: Body mass index
BP: Blood pressure
CDC: Centers for Disease Control
HEI: Healthy eating index
US: United States

## References

[1] Hales CM, Carroll MD, Fryar CD, Ogden CL (2017). Prevalence of obesity among adults and youth: United States, 2015–2016. NCHS Data Brief.

[2] Hales CM, Fryar CD, Carroll MD, Freedman DSOC (2018). Trends in obesity and severe obesity prevalence in US youth and adults by sex and age, 2007-2008 to 2015-2016. JAMA..

[3] Ogden CL, Lamb MM, Carroll MD, Flegal KM. Obesity and Socioeconomic Status in Children and Adolescents: United States*,* 2005–2008. Vol 127.; 2010. 21211166

[4] Halfon N, Larson K, Slusser W (2013). Associations between obesity and comorbid mental health, developmental, and physical health conditions in a nationally representative sample of US children aged 10 to 17. Acad Pediatr.

[5] Taras H, Potts-Datema W (2005). Obesity and student performance at school. J Sch Health.

[6] Barlow SE (2007). Expert Committee Recommendations Regarding the Prevention, Assessment, and Treatment of Child and Adolescent Overweight and Obesity: Summary Report. Pediatrics.

[7] Berge JM, Wall M, Hsueh T, Fulkerson JA, Larson N, Neumark-sztainer D (2015). The protective role of family meals for youth obesity: 10-year longitudinal associations. J Pediatr.

[8] Cason K (2006). Family mealtimes: more than just eating together. J Acad Nutr Diet.

[9] Fulkerson JA, Larson N, Horning M, Neumark-sztainer D (2014). A review of associations between family or shared meal frequency and dietary and weight status outcomes across the lifespan. J Nutr Educ Behav.

[10] Hammons AJ, Fiese BH (2011). Is frequency of shared family meals related to the nutritional health of children and adolescents?. Pediatrics..

[11] Larson NI, Neumark-Sztainer D, Hannan PJ, Story M (2007). Family meals during adolescence are associated with higher diet quality and healthful meal patterns during young adulthood. J Am Diet Assoc.

[12] Skafida V (2013). The family meal panacea: exploring how different aspects of family meal occurrence, meal habits and meal enjoyment relate to young children’s diets. Sociol Heal Illn.

[13] Videon TMC (2003). Influences on adolescent eating patterns: the importance of family meals. J Adolesc Health.

[14] Welsh EM, French SA, Wall M (2011). Examining the relationship between family meal frequency and individual dietary intake: does family cohesion play a role?. J Nutr Educ Behav.

[15] Dallacker M, Hertwig R, Mata J (2018). The frequency of family meals and nutritional health in children: a meta-analysis. Obes Rev.

[16] Fink SK, Racine EF, Mueffelmann RE, Dean MN, Herman-Smith R (2014). Family meals and diet quality among children and adolescents in North Carolina. J Nutr Educ Behav.

[17] Gable S, Chang Y, Krull JL (2007). Television watching and frequency of family meals are predictive of overweight onset and persistence in a national sample of school-aged children. J Am Diet Assoc.

[18] Rollins BY, Belue RZ, Francis LA (2010). The beneficial effect of family meals on obesity differs by race, sex, and household education: the National Survey of Children’s health, 2003-2004. J Am Diet Assoc.

[19] Woodruff SJ, Hanning RM, McGoldrick K, Brown KS (2010). Healthy eating index-C is positively associated with family dinner frequency among students in grades 6–8 from southern Ontario,Canada. Eur J Clin Nutr.

[20] Lee HJ, Lee SY, Park EC (2016). Do family meals affect childhood overweight or obesity?: nationwide survey 2008-2012. Pediatr Obes..

[21] Valdés J, Rodríguez-Artalejo F, Aguilar L, Jaén-Casquero MB, Royo-Bordonada MA (2013). Frequency of family meals and childhood overweight: a systematic review. Pediatr Obes..

[22] Fulkerson JA, Rydell S, Kubik MY (2010). Healthy home offerings via the mealtime environment (HOME): feasibility, acceptability, and outcomes of a pilot study. Obesity..

[23] Fulkerson JA, Neumark-Sztainer D, Story M (2014). The healthy home offerings via the mealtime environment (HOME) plus study: design and methods. Contemp Clin Trials.

[24] Fulkerson JA, Friend S, Flattum C, et al. Promoting healthful family meals to prevent obesity : HOME Plus , a randomized controlled trial. Int J Behav Nutr Phys Act. 2015:1–12. 10.1186/s12966-015-0320-3. 10.1186/s12966-015-0320-3 PMC4678662 26667110

[25] Dwyer L, Oh A, Patrick H, Hennessy E (2015). Promoting family meals: a review of existing interventions and opportunities for future research. Adolesc Health Med Ther.

[26] Bird Chris, Michie Colin (2008). Measuring blood pressure in children. BMJ.

[27] Fulkerson JA, Neumark-Sztainer D, Hannan PJ, Story M (2008). Family meal frequency and weight status among adolescents: cross-sectional and 5-year longitudinal associations. Obesity..

[28] Neumark-Sztainer D, Eisenberg ME, Fulkerson JA, Story M, Larson N (2008). Family meals and disordered eating. Arch Pediatr Adolesc Med.

[29] Neumark-Sztainer D, Wall M, Story M, Fulkerson JA (2004). Are family meal patterns associated with disordered eating behaviors among adolescents?. J Adolesc Health.

[30] Anderson LN, Lebovic G, Hamilton J (2016). Body mass index, waist circumference, and the clustering of Cardiometabolic risk factors in early childhood. Paediatr Perinat Epidemiol.

[31] Ramirez-Silva Ivonne, Rivera Juan A, Trejo-Valdivia Belem, Stein Aryeh D, Martorell Reynaldo, Romieu Isabelle, Barraza-Villarreal Albino, Avila-Jiménez Laura, Ramakrishnan Usha (2018). Relative Weight Gain Through Age 4 Years Is Associated with Increased Adiposity, and Higher Blood Pressure and Insulinemia at 4–5 Years of Age in Mexican Children. The Journal of Nutrition.

[32] Freemark M (2014). Predictors of childhood obesity and pathogenesis of comorbidities. Pediatr Ann.

[33] Birch L, Arbor A, Savage JS, Ventura A (2009). From infancy to adolescence. Can J Diet Pr Res.

[34] Hunter JG, Cason KL. Kids in the kitchen-. Clemson University Cooperative Extension- Home and Garden Information Center http://www.clemson.edu/extension/hgic/food/nutrition/nutrition/life_stages/hgic4113.html.

[35] Verhage CL, Gillebaart M, van der Veek SMC, Vereijken CMJL (2018). The relation between family meals and health of infants and toddlers: a review. Appetite..

[36] Rogers C, Anderson SE, Dollahite JS, et al. Methods and design of a 10-week multi-component family meals intervention: A two group quasi-experimental effectiveness trial. BMC Public Health. 2017;17(1). 10.1186/s12889-016-3908-x. 10.1186/s12889-016-3908-x PMC5223369 28069006

[37] Bartholomew LK, Parcel GS, Kok G (1998). Intervention mapping: a process for developing theory- and evidence-based health education programs. Heal EducBehav.

[38] Bandura A (1989). Social cognitive theory.

[39] Blumberg J, Bialostosky K, Hamilton WL, Briefel RR (1999). U.S. household food security survey module: six-item short form. Am J Public Health.

[40] Burrows TL, Martin RJ, Collins CE (2010). A systematic review of the validity of dietary assessment methods in children when compared with the method of doubly labeled water. J Am Diet Assoc.

[41] Bliss R (2004). Researchers produce innovation in dietary recall.

[42] University of Minnesota Nutrition Coordinating Center. Nutrition Data System for Research. 2015. http://www.ncc.umn.edu.

[43] Guenther P, Casavale K, Kirkpatrick S, Reedy J (2014). Update of the healthy eating index: HEI-2010. J Acad Nutr Diet.

[44] Lohman TG, Roche AF, Martorell R (1988). Anthropometric standardization reference manual.

[45] Centres for Disease Control and Prevention. Anthropometry procedures manual. *Natl Heal Nutr examinatory Surv*. 2007;(January):102. https://www.cdc.gov/nchs/data/nhanes/nhanes_07_08/manual_an.pdf%0Ahttp://www.cdc.gov/nchs/data/nhanes/nhanes_07_08/manual_an.pdf.

[46] Growth Charts - Z-score Data Files. CDC National Center for Health Statistics. https://www.cdc.gov/growthcharts/zscore.htm.

[47] Fryar CD, Gu Q, Ogden CL. Anthropometric reference data for children and adults: United States, 2007–2010. Vital Health Stat *11*. 2012;(252):1–48. http://www.ncbi.nlm.nih.gov/pubmed/25204692. Accessed January 28, 2019. 25204692

[48] Nhlbi. *The Fourth Report on the Diagnosis, Evaluation, and Treatment of High Blood Pressure in Children and Adolescents*. Bethesda, MD; 2004. https://www.nhlbi.nih.gov/files/docs/resources/heart/hbp_ped.pdf. 15286277

[49] Corporation IBM (2017). SPSS software 24.

[50] Statistical R. https://www.r-project.org/.

[51] Muzaffar H, Metcalfe JJ, Fiese B (2018). Narrative review of culinary interventions with children in schools to promote healthy eating: directions for future research and practice. Curr Dev Nutr.

[52] Utter J, Larson N, Laska MN, Winkler M, Neumark-Sztainer D (2018). Self-perceived cooking skills in emerging adulthood predict better dietary behaviors and intake 10 years later: a longitudinal study. J Nutr Educ Behav.

[53] Allirot X, da Quinta N, Chokupermal K, Urdaneta E (2016). Involving children in cooking activities: a potential strategy for directing food choices toward novel foods containing vegetables. Appetite..

[54] Méjean C, Lampuré A, Si Hassen W (2018). Influence of food preparation behaviors on 5-year weight change and obesity risk in a French prospective cohort. Int J Behav Nutr Phys Act.

[55] Clifford Astbury C, Penney TL, Adams J (2019). Comparison of individuals with low versus high consumption of home-prepared food in a group with universally high dietary quality: a cross-sectional analysis of the UK National Diet &amp; nutrition survey (2008–2016). Int J Behav Nutr Phys Act.

[56] Avery A, Anderson C, McCullough F. Associations between children’s diet quality and watching television during meal or snack consumption: A systematic review. Matern Child Nutr. 2017;13(4). 10.1111/mcn.12428. 10.1111/mcn.12428 PMC6866147 28211230

[57] Vik Frøydis N, Bjørnarå Helga, Øverby Nina C, Lien Nanna, Androutsos Odysseas, Maes Lea, Jan Natasa, Kovacs Eva, Moreno Luis A, Dössegger Alain, Manios Yannis, Brug Johannes, Bere Elling (2013). Associations between eating meals, watching TV while eating meals and weight status among children, ages 10–12 years in eight European countries: the ENERGY cross-sectional study. International Journal of Behavioral Nutrition and Physical Activity.

[58] Trofholz Amanda C., Tate Allan, Loth Katie, Neumark-Sztainer Dianne, Berge Jerica M. (2019). Watching Television while Eating: Associations with Dietary Intake and Weight Status among a Diverse Sample of Young Children. Journal of the Academy of Nutrition and Dietetics.

[59] Trofholz AC, Tate AD, Miner MHBJ (2017). Associations between TV viewing at family meals and the emotional atmosphere of the meal, meal healthfulness, child dietary intake, and child weight status. Appetite..

[60] Kasper N, Mandell C, Ball S, Miller AL, Lumeng J, Peterson KE (2016). The healthy meal index: a tool for measuring the healthfulness of meals served to children. Appetite..

[61] Sexton K, Greaves IA, Church TR (2010). A school-based strategy to assess children's environmental exposures and related health effects in economically disadvantaged urban neighborhoods. J Expo Sci Environ Epidemiol.

[62] Boushey C J, Kerr D A, Wright J, Lutes K D, Ebert D S, Delp E J (2009). Use of technology in children’s dietary assessment. European Journal of Clinical Nutrition.

[63] Allman-Farinelli M, Gemming L (2017). Technology interventions to manage food intake: where are we now?. Curr Diab Rep.

[64] Casperson SL, Sieling J, Moon J, Johnson L, Roemmich JN, Whigham L (2015). A Mobile phone food record app to digitally capture dietary intake for adolescents in a free-living environment: usability study. JMIR mHealth uHealth.

[65] Hunter J, Cason K (2008). Kids in the kitchen.

[66] McGowan HM, Nix RL, Murphy SA, Bierman KL (2010). Investigating the impact of selection bias in dose-response analyses of preventive interventions. Prev Sci.

[67] Pyatak EA, Carandang K, Vigen CLP (2018). Occupational therapy intervention improves glycemic control and quality of life among young adults with diabetes: the resilient, empowered, active living with diabetes (REAL diabetes) randomized controlled trial. Diabetes Care.

[68] Fulkerson JA, Farbakhsh K, Lytle L (2011). Away-from-home family dinner sources and associations with weight status, body composition, and related biomarkers of chronic disease among adolescents and their parents. J Am Diet Assoc.

[69] Davis JN, Ventura EE, Cook LT, Gyllenhammer LE, Gatto NM (2011). LA sprouts: a gardening, nutrition, and cooking intervention for Latino youth improves diet and reduces obesity. J Am Diet Assoc.

[70] Pastor-Barriuso Roberto, Banegas Jos R., Damin Javier, Appel Lawrence J., Guallar Eliseo (2003). Systolic Blood Pressure, Diastolic Blood Pressure, and Pulse Pressure: An Evaluation of Their Joint Effect on Mortality. Annals of Internal Medicine.

[71] Fruh SM, Mulekar MS, Crook E, Hall HR, Adams J, Lemley T (2018). The family meal challenge. J Christ Nurs.

[72] Anderson JD, Newby R, Kehm R, Barland P, Hearst MO (2015). Taking steps together: a family- and community-based obesity intervention for urban, Multiethnic Children. Heal Educ Behav.

[73] Davis DS, Goldmon MV, Coker-Appiah DS (2011). Using a community-based participatory research approach to develop a faith-based obesity intervention for African American children. Health Promot Pract.

[74] Lutz A. Cost to eat at every major fast food chain - Business Insider. Business Insider. https://www.businessinsider.com/cost-to-eat-at-every-major-fast-food-chain-2015-9?IR=T. Published 2015.

[75] Kneipp SM, Lutz BJ, Levonian C, Cook C, Hamilton JB, Roberson D (2013). Women’s experiences in a community-based participatory research randomized controlled trial. Qual Health Res.

[76] Mode NA, Evans MK, Zonderman AB (2016). Race, neighborhood economic status, income inequality and mortality. PLoS One.

[77] Foster E, Bradley J (2018). Methodological considerations and future insights for 24-hour dietary recall assessment in children. Nutr Res.

[78] Gibson RS, Charrondiere UR, Bell W (2018). Measurement errors in dietary assessment using self-reported 24-hour recalls in low-income countries and strategies for their prevention. Adv Nutr An Int Rev J.

